# Comparative Expression Profiling of Distinct T Cell Subsets Undergoing Oxidative Stress

**DOI:** 10.1371/journal.pone.0041345

**Published:** 2012-07-20

**Authors:** Rudolf Lichtenfels, Dimitrios Mougiakakos, C. Christian Johansson, Sven P. Dressler, Christian V. Recktenwald, Rolf Kiessling, Barbara Seliger

**Affiliations:** 1 Institute of Medical Immunology, Martin Luther University Halle-Wittenberg, Halle, Germany; 2 Department of Oncology and Pathology, Cancer Center Karolinska, Stockholm, Sweden; 3 Department of Internal Medicine 5, Hematology and Oncology, University of Erlangen-Nuremberg, Erlangen, Germany; New York University, United States of America

## Abstract

The clinical outcome of adoptive T cell transfer-based immunotherapies is often limited due to different escape mechanisms established by tumors in order to evade the hosts' immune system. The establishment of an immunosuppressive micromilieu by tumor cells along with distinct subsets of tumor-infiltrating lymphocytes is often associated with oxidative stress that can affect antigen-specific memory/effector cytotoxic T cells thereby substantially reducing their frequency and functional activation. Therefore, protection of tumor-reactive cytotoxic T lymphocytes from oxidative stress may enhance the anti-tumor-directed immune response. In order to better define the key pathways/proteins involved in the response to oxidative stress a comparative 2-DE-based proteome analysis of naïve CD45RA^+^ and their memory/effector CD45RO^+^ T cell counterparts in the presence and absence of low dose hydrogen peroxide (H_2_O_2_) was performed in this pilot study. Based on the profiling data of these T cell subpopulations under the various conditions, a series of differentially expressed spots were defined, members thereof identified by mass spectrometry and subsequently classified according to their cellular function and localization. Representative targets responding to oxidative stress including proteins involved in signaling pathways, in regulating the cellular redox status as well as in shaping/maintaining the structural cell integrity were independently verified at the transcript and protein level under the same conditions in both T cell subsets. In conclusion the resulting profiling data describe complex, oxidative stress-induced, but not strictly concordant changes within the respective expression profiles of CD45RA^+^ and CD45RO^+^ T cells. Some of the differentially expressed genes/proteins might be further exploited as potential targets toward modulating the redox capacity of the distinct lymphocyte subsets thereby providing the basis for further studies aiming at rendering them more resistant to tumor micromilieu-induced oxidative stress.

## Introduction

Oxidative stress- and activation-induced cell death of memory cytotoxic T cells is not only associated with chronic inflammation, but also represents an important immune escape mechanism in cancer as a consequence of shaping an immunosuppressive micromillieu towards mimicking inflammatory conditions. Reactive oxygen species (ROS) and/or reactive nitrogen species (RNS) are produced and released in high amounts by malignant cells as well as by tumor-infiltrating bystander cells like activated granulocytes, tumor-associated macrophages (TAM) and myeloid-derived suppressor cells (MDSC) [1]. Both, ROS and RNS promote tumor growth, angiogenesis and metastases formation [2]–[3] as bona fide second messengers as well as by hampering immune responses, e.g. through functional alterations and/or the induction of apoptosis [1], [4].

So far, oxidative stress-induced immune suppression has been investigated in several models although the complete mechanisms and pathways involved in T cell hypo-responsiveness remain undetermined [5]. There exist some selected studies investigating the functional consequences of ROS for various immune cells, including T cells and dendritic cells (DC). The relevant effects were studied within co-culture experiments using activated macrophages/granulocytes as sources of NO or H_2_O_2_ or upon treatment with exogenous H_2_O_2_, respectively. Co-culturing T cells with tumor-associated macrophages (TAM) resulted in a decreased expression of the T cell receptor (TCR) zeta chain and a loss of antigen-specific cells. Various immune cell populations including T [6], [7], [8], [9], [10] and NK [11], [12], [13] cell subsets have been analyzed in regards to their sensitivity towards ROS. Treatment of peripheral blood lymphocytes (PBL) or purified T cell subsets at concentrations >25 µM H_2_O_2_ selectively targets the cytokine production of CD45RO^+^ memory/activated T cells [7]. The loss of Th1 cytokine production in CD45RO^+^ T cells, in particular of tumor necrosis factor (TNF)-alpha, interferon (IFN)-gamma and interleukin (IL)-2, was associated with the inhibition of the nuclear factor kappa B (NF-kB) activation/nuclear translocation and subsequently with a decrease in the NF-kB-mediated cytokine transcription rate [7]. Furthermore, H_2_O_2_ interferes in CD45RO^+^ T cells with a proper signaling downstream of the TCR by blocking the important regulatory protein tyrosine phosphatases (PTP). In contrast, exposures to lower H_2_O_2_ concentrations only marginally affected the cytokine secretion pattern except for IL-10, which was still reduced in both T cell subsets, but independent from blocking NF-kB activation. Time kinetic experiments revealed an increase in both early (annexin V^+^, 7-AAD^−^) and late (annexin V^+^, 7-AAD+) apoptotic cells in the CD45RO+ T cell subset after 6 hrs, but not after 3 hrs of treatment with 5 µM H_2_O_2_, [10]. However, the short term exposure resulted in a loss of T cell function without significant apoptosis providing evidence that there is a dose window of exposure to oxidative stress in which cells enter an anergic rather than a pre-apoptotic state [10], thereby defining the treatment conditions for the proteomic profiling in the present study. Central memory and effector memory T cells have been shown to be more sensitive to treatment with low doses of H_2_O_2_ than their naïve counterparts [10], whereas regulatory T cells (Tregs) revealed a high anti-oxidative capacity [8] comparable to that of CD56^bright^ NK cells and thus maintain their suppressive function under oxidative stress [13].

Low dose H_2_O_2_ can interfere with the mitochondrial pathway of apoptosis in CD45RO^+^ T cells [10]. The induction of suppressors of cytokine signaling (SOCS) and activation of Janus kinase (JAK) represent important inhibitors of the ROS-induced apoptosis [14]. However, a better knowledge about the altered key pathways during oxidative stress in T cell subsets might allow to rescue these important effectors in the future by either genetic and/or pharmacologic interventions thereby significantly bolstering their therapeutic efficacy.

In addition to the decreased CD3 zeta chain expression the molecular mechanisms underlying the ROS-induced signaling defects in hypo-responsive immune cells include the down-modulation of the tyrosine protein kinase Lck (p56Lck) and of the linker for activation of T cells family member 1 (LAT) [15]. Since the phosphoinositol-3-kinase (PI3K) signaling pathway is not influenced by exposure to H_2_O_2_ oxidative stress in T cells appears to be rather mediated by other signal transduction pathways. Moreover, it might be countered by differential protein expression or altered protein degradation pattern in particular of enzymes involved in maintaining the cellular redox capacity. The complex regulation of the cellular redox system affects immune responses in a dual fashion, where H_2_O_2_ as the main representative of ROS acts either as a (activating) second messenger or as a negative regulator of immune reactivity. Indeed, proteome-based analyses have recently demonstrated an association of aberrant ROS signaling with autophagy [16]. In order to shed further light onto these processes, this report aimed at identifying markers and molecular pathways that characterize the response of CD45RO^+^/RA^+^ T cell subsets to oxidative stress. Thus, comparative two-dimensional gel electrophoresis (2-DE)-based proteome analysis followed by matrix-assisted laser desorption time of flight mass spectrometry (MALDI-TOF-MS)-based protein identification and subsequent bioinformatic analyses were performed in order to determine the complex effects of H_2_O_2_ on the protein expression pattern in CD45RA^+^/RO^+^ T cells. Our findings may lead to a better understanding of oxidative-stress related molecules, which at the same time might also represent promising targets to strengthen the cellular resistance towards oxidative stress-induced cell death and suggest that the response to oxidative stress depends on the utilization of several scavenger pathways, but not in a hierarchical order.

## Results

### Immune cell enrichment and proof of treatment efficacy

**Figure 1:**
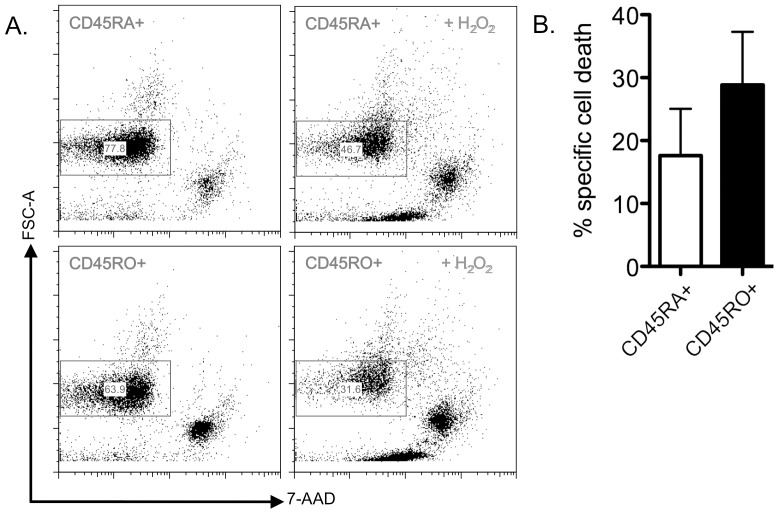
Representative cell viability in response to treatment with low doses of H2O2. A. Representative FACS data for untreated vs. H_2_O_2_-exposed (5 µM} T cell subsets isolated from one healthy donor are shown. Cell viability (gate) was assessed by changes of the forward scatter (FSC-A) profile and staining with 7-AAD.. B. Mean values for specific H_2_O_2_-induced cell death are shown (n = 5). Bars represent the standard error mean.

A prerequisite for the proteome analysis is an efficient immune cell enrichment in order to obtain sufficient and pure amounts of T cell subpopulations. Using a pan T cell selection kit in combination with anti-CD45RA− or anti-CD45RO-specific beads, naïve CD45RA^+^ and memory CD45RO^+^ T cells were isolated with a purity of >95% (Supplementary Figure S1). Moreover, the efficacy of the treatment with low doses of H_2_O_2_ (5 µM) was monitored by assessing cell viability by flow cytometry using 7-AAD stainings of the distinct T cell subsets as representatively shown in Figure 1. Figure 1A shows a drop in cell viability in response to treatment with 5 µM H_2_O_2_ and subsequent culturing of the cells for >24 h in serum free medium from 77.8% to 46,7% in the subset of CD45RA^+^ T cells (upper panels) of a representative donor. The effect on the CD45RO^+^ T cells (lower panels) is even stronger, resulting in the survival of merely 50% of the cells. Figure 1B shows the overall assessment of the cell viability within the respective T cell subsets. The mean cell death rates in response to exposure to 5 µM H_2_O_2_ as defined in 5 individual donors slightly differs between the two distinct subsets reaching about 18% for CD45RA^+^ T cells and 28% for CD45RO^+^ T cells, respectively. Moreover, the H_2_O_2_-induced cell death following culturing for >24 h in serum free medium was significant for both CD45RA^+^ (p = 0.04) and CD45RO^+^ (p = 0.02) T cell subsets.

### Differential protein expression pattern in the different immune cell populations

**Figure 2:**
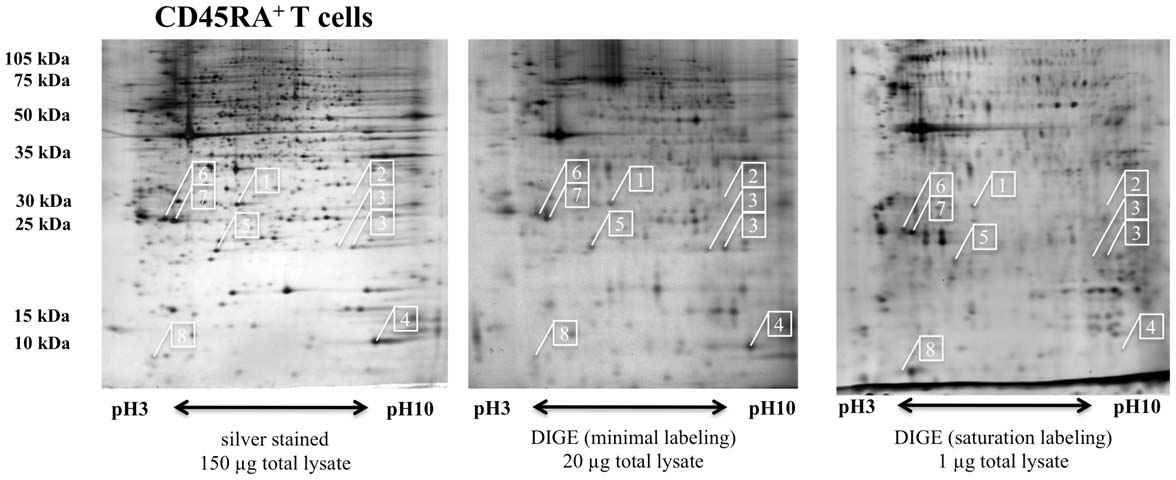
Representative protein expression pattern of CD45RA^+^ T cells using distinct staining methods. Representative protein expression profiles of CD45RA^+^ T cells post separation by two-dimensional gel electrophoresis in 16% T, 2.5% C polyacrylamide gels, The staining procedures (A) silver staining, (B) DIGE minimal staining and (C) DIGE saturating staining were performed as outlined in the Materials an methods section. The gels were loaded with 150 µg, 20 µg or 1 µg of total lysate, respectively. The numbers boxed in white squares indicate the relative localization for the panel of selected differentially expressed targets (1 – GSTO1, 2 – LIMS1, 3 – SODM, 4 – PROF1, 5 – PRDX2, 6 – GDIR1, 7 – GDIR2 and 8 – THIO). The pH gradient used in the first dimension (x axis) as well as the range of the size fractionation achieved in the second dimension (y axis) are indicated.

The purified CD45RA^+^ and CD45RO^+^ T cells either cultured in the absence or presence of 5 µM H_2_O_2_ were subjected to 2DE-based proteome analysis applying different staining/labeling protocols. Whereas the initial protein expression pattern was established based on silver stainings of 2–3 technical replicates per sample, additional comparative expression profilings of 4 independent sample sets applying minimal or saturating difference gel electrophoresis (DIGE) labelings were performed to track biological variances within the respective expression pattern. The total spot numbers/gel in the different experimental settings varied between 1004 and 1179 spots for CD45RA^+^ (naïve) and CD45RO^+^ (memory) T cells (Table 1). As representatively shown in Figure 2 for CD45RA^+^ T cells distinct, but largely stable protein expression pattern independently of the applied staining/labeling technologies could be established for both T cell subsets.

**Table 1:** Number of matched and regulated protein spots in CD45RA^+^ and CD45RO^+^ T cell subsets using distinct staining technologies.

staining method	silver staining				DIGE (minimal labeling)				DIGE (saturating labeling)			
sample	RA−	RA+	RO−	RO+	RA−	RA+	RO−	RO+	RA−	RA+	RO−	RO+
total no. of spots	1053	1082	1004	1179	1144	1144	1084	1051	1102	1100	1102	1101
no. of matched spots	765		837		891		836		1102		1102	
no. of upreg. spots	72		127		71		50		230		354	
no. of downreg. spots	57		103		92		80		469		252	
no. of not reg. spots	636		607		728		702		394		496	

RA− and RO− indicate the profiling results for the respective untreated T cell subsets, RA+ and RO+ for that of H_2_O_2_-treated T cell subpopulation as outlined in the Material and methods section. Moreover, the profiling data for the pattern resulting from silver stainings/minimal DIGE labelings were established with the software package Proteomweaver 4.0 as outlined in the Material and methods section.

### Comparative expression profiling of CD45RA^+^ vs. CD45RO^+^ T cells

Based on the initial comparative expression profiling of CD45RA^+^ vs. CD45RO^+^ T cells 40 differentially expressed protein spots were selected and subsequently subjected to mass spectrometry. From the subset of 33 up-regulated spots 30 provided analytical data, leading to the identification of 20 single target spots each representing a single protein species. In addition 10 spots that contained more than a single protein species were classified as multiple target spots, whereas the protein content in the remaining 3 spots was below the detection limit, respectively. Several of the identified proteins, including the heat shock cognate 71 kDa protein (HSP7C), the fibrinogen beta chain (FIBB) and the fructose-bisphosphate aldolase A (ALDOA) were represented in more than 1 spot within the respective protein expression profiles suggesting that these proteins had undergone posttranslational modifications (PTM), although their nature has not yet been defined. From the selected panel of 7 down-regulated spots, 3 provided interpretable analytical data all representing single target spots, but the protein content of the remaining spots were again below the detection limit of the used mass spectrometer. The majority of the identified proteins represent (i) components of the cytoskeleton such as actins (ACTB/ACTG), tubulins (tubulin alpha (TBA1A, TBA4A) and tubulin beta (TBB5), talin-1 (TLN1), vinculin (VINC) or profilin-1 (PROF1), (ii) proteins involved in the cellular energy metabolism, e.g. alpha-enolase (ENOA), phosphoglycerate kinase 1 (PGK1), ALDOA or the mitochondrial ATP synthase subunit beta (ATPB), but also (iii) scavenger antioxidants, like manganese superoxide dismutase (SODM) or thioredoxin 1 (THIO) and (iv) members of signaling pathways including the LIM and senescent cell antigen-like domain containing protein 1 (LIMS1) and the SH3 domain-binding glutamic acid–rich-like protein 3 (SH3L3) (Table S1, column 3).

### Comparative expression profiling of untreated vs. H_2_O_2_-treated CD45RA^+^ and/or CD45RO^+^ T cells

In order to define differences in the cellular response to low concentration of H_2_O_2_ (5 µM) between the two distinct T cell subsets a total number of 94 protein spots defined as differentially expressed upon H_2_O_2_-treatment were selected for identification by mass spectrometry. From these 80 provided interpretable spectra, while the remaining 14 protein spots were below the detection limit of the used mass spectrometer. By eliminating spots containing more than one protein species the number of differentially expressed proteins was reduced to 63 single target spots, each representing an individual protein identity (Table S1, columns 1, 2 and 4).

The classification of these differentially expressed proteins according to their cellular function based on their gene ontology (GO) annotations revealed that 25% of these proteins exhibit structural functions, 22% are associated with the cellular metabolism, 14% each are linked to signal transduction processes or cellular stress, 7% exert multiple functions and 5% are linked to transport processes. The remaining 13% of proteins were classified as “others” represent a pool of proteins involved in functions with a <5% frequency (Figure S2A,). Concerning their cellular localization the proteins are predominantly localized in the cytoplasm (28%), in multiple compartments (27%) or in the cytoskeleton (16%). Less frequently represented are proteins derived from mitochondria, secreted proteins (each at 9%) or from other compartments (“others”) again comprised of compartments represented at a frequency <5% (Figure S2B).

Some of the differentially expressed proteins representing members of the major functional categories listed in Table 2 (Figure S2A) that are either directly or indirectly linked to relevant signaling pathways, such as the Rho GDP-dissociation inhibitor 1 (GDIR1) and Rho GDP-dissociation inhibitor 2 (GDIR2), to the response to oxidative stress, like (SODM, peroxiredoxin 2 (PRDX2), thioredoxin 1 (THIO) and glutathione-A-transferase omega 1 (GSTO1), to the regulation of cell survival (LIMS) or to the stabilization/destabilization of the cytoskeleton (PROF1) were selected for further verification (Table 3). A comprehensive summary of the 2-DE-based profiling results for the panel of targets selected for further validation studies across the various biological replicates is provided in Table 4.

**Table 2:** Functional classification of differentially expressed proteins.

match set function	RO− vs. RA−RO+ vs. RA+	RA+ vs. RA−	RO+ vs. RO−
up-regulated	TBA1A, TBA4A, TBA8A, TLN1, VINC	TBA4A, TPM1, TPM3, TPM4, ARPC2, ACTN1, FLNA, TLN1, VINC, VIME, PLSL	TBB5. TBA4A, FLNA, VINC, COF1, ARP3
structural molecules			
down-regulated	TBB5, TPM4, PROF1	ACTN1, TPM1, TPM2,VIME	TPM1, FLNA
up-regulated	ALDOA, TPIS, KPYM, ENOA, TKT, ATPB, ATP5H	ENOA	ATPB, ATP5H
energy metabolism			
down-regulated		ALDOA, PKM2	KPYM, LDHB, TALDO, ATP5H
up-regulated	SODM, G6PD	GSTO1, PRDX2	
redox status			
down-regulated	THIO		SODM, SH3L3
up-regulated	ANXA5, LIMS1	GDIR1, ITA2B, 1433Z	GDIR2
signaling			
down-regulated		TSP1, LIMS1	GRB2
up-regulated	GRP78, HSP7C, PDIA3	HS90A, CH60, GRP78, HSP7C	HS90A, GRP78, HSP7C, GRP75, PDIA3
cell stress, protein folding			

RA− and RO− indicate the profiling results for the respective untreated T cell subsets, RA+ and RO+ for that of H_2_O_2_-treated T cell subpopulation as outlined in the Material and methods section. Proteins are listed by their protein entry names.

**Table 3:** Relevant characteristics and MALDI peptide fingerprinting data for the subset of verified proteins.

Protein entry name	SwissProt ID	MW (Da)	MW (Da) 2-DE	seq. cov.	Mascot score	match set silver staining profile
GSTO1	P78417	27833	30000	32%	72	CD45RA+ vs. CD45RA−, up-reg
PRDX2	P32119	21918	25000	38%	103	CD45RA+ vs. CD45RA−, up-reg.
PROF1	P07737	15085	15000	54%	76	CD45RO vs. CD45RA, down-reg.
LIMS1	P48059	38977	35000	29%	83	CD45RO vs. CD45RA, up-regCD45RA+ vs. CD45RA−, down-reg
GDIR1^*^	P52565	23119	28000	47%	103	CD45RA+ vs. CD45RA−, up-reg.
GDIR2	P52566	22900	28000	56%	75	CD45RO+ vs. CD45RO−, up-reg
SODM	P04179	24878	23000	47%	125	CD45RO vs. CD45RA, up-regCD45RO+ vs CD45RO−, down-reg
THIO^*^	P10599	11884	13000	49%	51	CD45RO vs. CD45RA, down-reg.

RA− and RO− indicate the profiling results for the respective untreated T cell subsets, RA+ and RO+ for that of H_2_O_2_-treated T cell subpopulation as outlined in the Material and methods section. Proteins are listed by their protein entry names. The * indicates, that the MALDI data for these targets were previously published in an independent proteomic profiling study of T cells using the same set of preparative gels for target identification [44].

**Table 4:** Combined expression pattern analysis for the panel of validated targets in independent 2-DE analyses.

Protein entry name	DIGE 1 (min)	DIGE 2 (sat)	DIGE 3 (sat)	DIGE 4 (sat)
	RA+/RA−	RA+/RA−	RA+/RA−	RA+/RA−
GSTO1	0.6	0.2	0.4	0.4
LIMS1	0.9	0.8	0.4	0.6
SODM	0.8/0.9	1.7/0.7	9.3/2.3	0.5/1.3
PROF1	1.0	0.6	0.8	n.d.
PRDX2	1.0	0.6	1.2	0.4
GDIR1	1.1	2.0.	3.6	0.9
GDIR2	1.1	2.1	2.3	0.7
THIO	1.0.	0.7	6.6	0.4

	RO+/RO−	RO+/RO−	RO+/RO−	RO+/RO−
GSTO1	1.0	1.5	2.1	4.0
LIMS1	0.9	0.9	14.7	48
SODM	0.9/1.0	0.7/1.3	1.1/0.9	0.8/1.1
PROF1	1.0	3.5	1.6	n.d.
PRDX2	0.9	0.5	0.5	0.5
GDIR1	0.8	0.6	0.4	0.8
GDIR2	1.2	0.6	0.8	0.6
THIO	0.5	0.7	0.7	4.0

RA− and RO− indicate the profiling results for the respective untreated T cell subsets, RA+ and RO+ for that of H_2_O_2_-treated T cell subpopulation as outlined in the Material and methods section. Proteins are listed by their protein entry names. n.d. stands for not detectable in one or both samples; not reg. for not regulated, up-reg. for up-regulated, down-reg. for down-regulated and het. reg. for heterogeneously regulated. For SODM 2 variants were identified.

### Verification of the 2-DE-based differential expression pattern for representative targets as defined in the respective comparisons of the two distinct CD45^+^ T cell subsets in the presence or absence of H_2_O_2_

**Figure 3:**
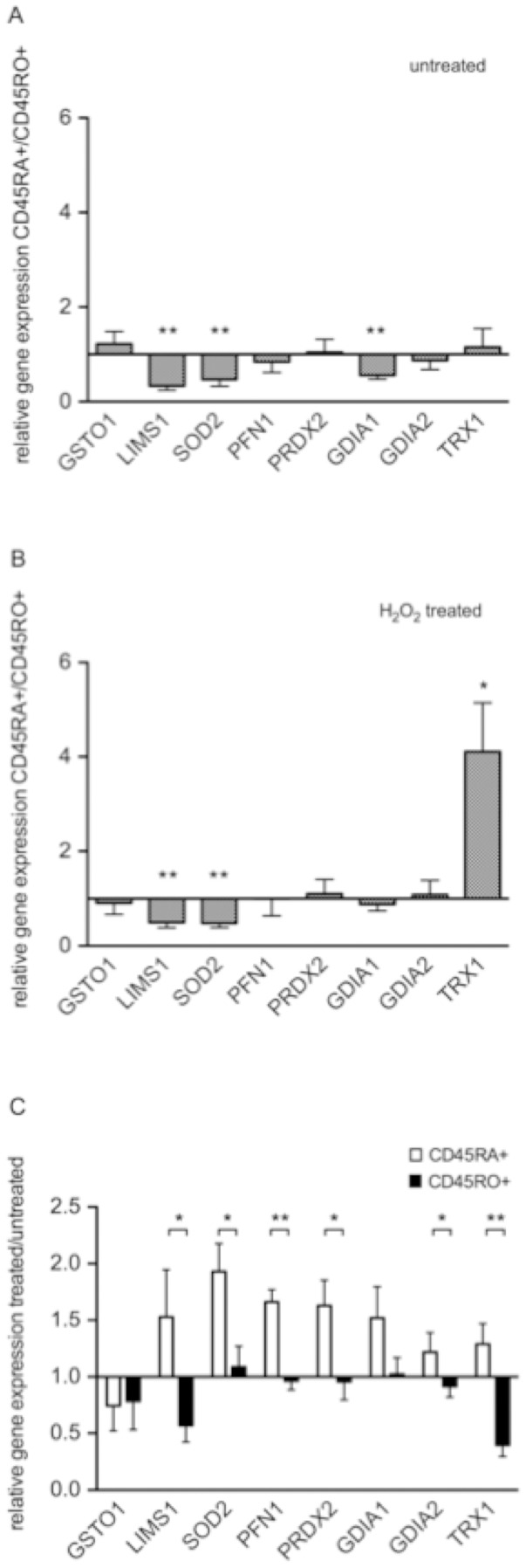
Comparative mRNA expression profiling for the selected panel of differentially expressed targets in the various T cell subsets. Up-/down-regulation of the relative gene expression levels for the selected members of differentially expressed target structures in (A) untreated CD45RA^+^ vs. untreated CD45RO^+^ T cells; (B) H_2_O_2_-treated CD45RA^+^ vs. H_2_O_2_-treated CD45RO^+^ T cells and (C) H_2_O_2_-treated vs. untreated naïve CD45RA^+^ (white bars) or memory/effector CD45RO^+^ (black bars) T cells (n = 6). T cells were either left untreated or treated with 5 µM H_2_O_2_ for 3 hours as described in the Materials and methods section. The error bars represent the given standard error of the mean values. P-values <0.05 are marked with a star, whereas p-values <0.01 are marked with 2 stars.

The differential expression pattern for a selected panel of 8 representative targets was verified at the transcriptional level by performing qRT-PCR analyses using identically treated naïve and memory/effector CD45^+^ T cell subsets obtained from 6 healthy donors. The comparative mRNA expression profilings of untreated and H_2_O_2_-treated CD45RA^+^ and CD45RO^+^ T cells (Figure 3A, 3B) revealed an up-regulation of LIMS1, manganese superoxide dismutase (SOD2) and Rho GDP-dissociation inhibitor 1 (GDIA1) in CD45RO^+^ T cells (Figure 3A). The most striking difference between the naïve and memory/effector CD45^+^ T cell subsets under oxidative stress conditions was observed for thioredoxin 1 (TRX1), which is significantly down-regulated in the CD45RO^+^ T cell subset (Figure 3B).. Furthermore, the side by side comparison of untreated vs. H_2_O_2_-exposed T cells for the distinct subsets (Figure 3C) reveals that GSTO1 is down-regulated at the transcriptional level in both subsets under oxidative stress, whereas LIMS1 and TRX1 are up-regulated in CD45RA^+^, but down-regulated in H_2_O_2_-treated CD45RO^+^ T cells. Moreover, SOD2, profilin 1 (PFN1), PRDX2, GDIA1 and GDIA2 mRNA levels were up-regulated in H_2_O_2_-exposed CD45RA^+^ T cells, but not in CD45RO^+^ T cells, which show rather stable transcript levels for these target genes.

Thus, the transcriptomic profiling data largely support the proteomic profiling results, such as the up-regulation of LIMS1 and SODM/SOD2 and the down-regulation of THIO/TRX1 observed in the comparison between the CD45RO^+^ and CD45RA^+^ T cell subsets. Interestingly, the down-regulation of PROF1 seems to be mRNA-independently regulated since the corresponding PFN1 levels remained unchanged. The latter phenomena was also observed for the target SODM in CD45RO^+^ T cells undergoing oxidative stress and for GDIR2 up-regulated in the same system based on the proteomic profiling, but not at the corresponding transcript level. The up-regulation of the transcription rates of PRDX2 and GDIR1 in CD45RA^+^ T cells undergoing oxidative stress as well as the up-regulation of the transcript levels of LIMS1 and SODM observed in the direct comparison between the two H_2_O_2_-treated T cell subsets are in line with the proteomic profiling data. In contrast, an inverse regulation pattern was found for the target GSTO1 up-regulated in the proteomic profile, but down-regulated at the transcription level and for LIMS1 regulated vice versa.

**Figure 4:**
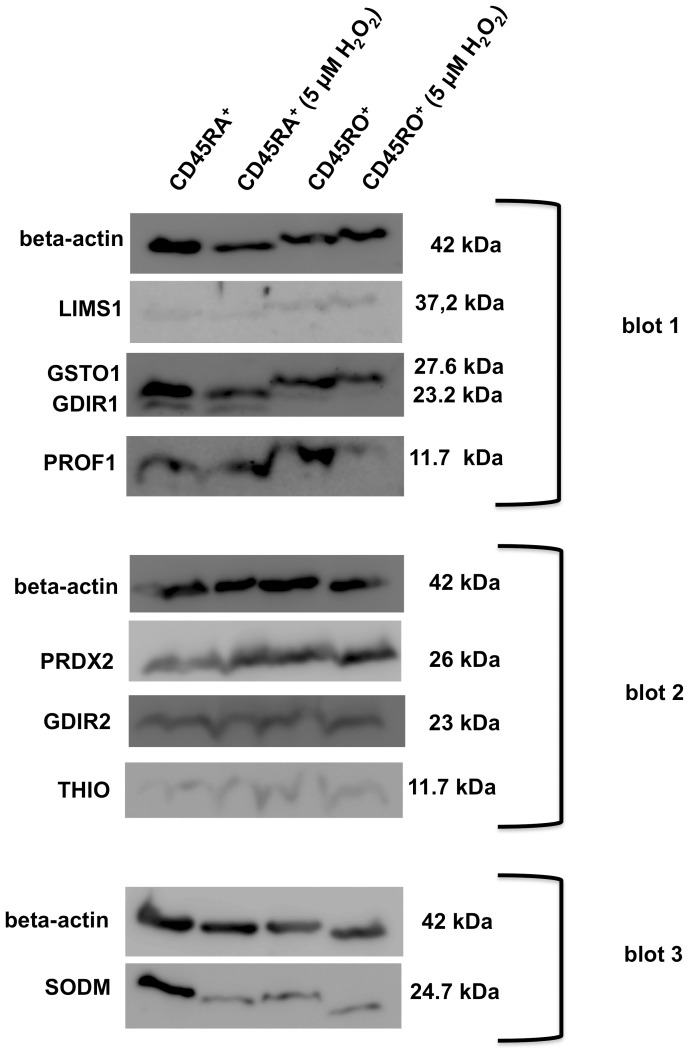
Representative Western blot analysis for the selected panel of differentially expressed targets in the various T cell subsets. Western blot analyses were performed by serially probing sets of 3 membranes per donor as outlined in the Material and methods section. The expression levels of GDIR1, GSTO1, LIMS1 and PROF1 were analyzed blot 1 (upper panel), GDIR2, PRDX2 and THIO on blot 2 (middle panel) and SODM on blot 3 (lower level) of the respective membrane set. The relative expression levels were defined by densitometric quantification normalized to the corresponding beta-actin signals, co-detected on each membrane. Aliquots of 15 µg total lysate per sample/lane were loaded onto Tris-Tricine gels (16%T/2.5%C) and subsequently separated and processed as outlined in the Materials and method section. Lysates generated form untreated CD45RA^+^ and CD45RO^+^ T cells were throughout the set of membranes loaded onto the lanes 1 and 3 respectively, whereas lysates generated from the corresponding T cell subsets exposure for 3 h to 5 µM H_2_O_2_ were loaded onto the lanes 2 and 4 as indicated. The protein entry names of the targets (left) and their corresponding molecular weight (right) are listed.

The 2-DE-based differential expression pattern defined in the respective comparisons of the two distinct CD45^+^ T cell subsets in the presence or absence of H_2_O_2_ was also verified for the same set of representative targets also at the protein level by performing semi-quantitative Western blot analyses from samples obtained from at least 3 independent donors (Table 5). Per donor sets of 3 technical replicate membranes were generated and subsequently serially probed with the respective target-specific antibodies, thereby preventing membrane strippings. As representative shown in Figure 4 the relative expression levels of GDIR1, GSTO1, LIMS1 and PROF1 were determined per donor-specific membrane set on blot 1 (upper panel), GDIR2, PRDX2 and THIO on blot 2 (middle panel) and SODM blot 3 (lower panel)..

**Table 5:** Combined expression pattern analysis for the panel of validated targets in independent Western blot analyses.

Protein entry name	Donor 1	Donor 2	Donor 3	Donor 4	Donor 5
	RA+/RA−	RA+/RA−	RA+/RA−	RA+/RA−	RA+/RA−
GSTO1	2.6	1.4	1.0	1.2	1.5
LIMS1	0.8	1.2	1.1	0.5	1.2
SODM	1.0	0.7	1.2	0.6	0.4
PROF1	1.1	2.4	n.d.	1.3	n.d.
PRDX2	1.6	0.3	1.0	0.9	1.0
GDIR1	n.d.	n.d.	4.9	1.0	1.7
GDIR2	1.7	0.6	1.2	1.3	2.3
THIO	n.d.	0.6	n.d.	0.7	0.6

	RO+/RO−	RO+/RO−	RO+/RO−	RO+/RO−	RO+/RO−
GSTO1	2.8	2.6	0.7	0.4	2.1
LIMS1	2.0	2.4	1.0	0.6	1.6
SODM	1.5	0.7	0.7	0.7	0.5
PROF1	2.2	1.8	n.d.	0.4	n.d.
PRDX2	1.4	1.7	0.6	1.7	1.1
GDIR1	n.d.	n.d.	1.0	0.7	1.0
GDIR2	1.9	1.0	0.2	1.4	0.2
THIO	2.0	1.0	n.d.	1.4	n.d.

RA− and RO− indicate the profiling results for the respective untreated T cell subsets, RA+ and RO+ for that of H_2_O_2_-treated T cell subpopulation as outlined in the Material and methods section. Proteins are listed by their protein entry names. n.d. stands for not detectable in one or both samples; not reg. for not regulated, up-reg. for up-regulated, down-reg. for down-regulated and het. reg. for heterogeneously regulated.

The up-regulation of GSTO1 and GDIR1 in CD45RA^+^ T cells under oxidative stress as defined by the 2-DE-based proteomic profiling is strongly supported by the Western blot results (Table 5), whereas for PRDX2 and LIMS1 rather heterogeneous regulation pattern were detected suggesting that these targets are less consistently regulated. However, some of the donors such as donor 1 donor 4 show a similar expression pattern for PRDX2 and LIMS1 as defined by the initial proteomic profiling. The down-regulation of SODM in H_2_O_2_-treated CD45RO^+^ T cells is also supported by the Western blot analysis (Table 5). I contrast, GDIR2 up-regulated in the proteomic profiling exerted a heterogeneous donor-dependent regulation pattern in the Western blot analyses suggesting that the expression level of GDIR2 is not strictly correlated with the exposure to H_2_O_2_ (Table 5).. In addition, the obtained Western blot profiling results also support the increased expression of THIO as well as the reduced expression of SODM and LIMS1 in CD45RA^+^ T cells as initially defined in the direct comparison of the 2 untreated T cell subsets.

## Discussion

Oxidative stress results in changes of the mRNA and protein expression pattern in mammalian cells. For the neutralization of ROS cells are equipped with intracellular reducing agents such as enzymes of the oxidant trap like SODM, catalase (CAT), glutathione peroxidases (GPX) and THIO [17] or non-enzymatic antioxidants like carotenes, coenzyme Q 10, glutathione (GSH), lipoic acid, vitamin C or vitamin E [18], which all might contribute to inactivate ROS. Furthermore, they can trap electrophilic compounds by conjugation of GSH or glucuronic acid via glutathione-S-transferases (GSTs) or UDP-glucuronosyl-transferases subsequently facilitating their excretion.

Under pathophysiologic conditions oxidative stress is modulating proteins through oxidation of thiol groups leading to alterations of the expression level, cellular localization and/or function/signaling [19], [20], [21]. Thus, reactive oxygen species (ROS) as components of the immune suppressive tumor microenvironment might also promote neoplastic transformation and/or tumor progression.. Although oxidation occurs rather randomly throughout all protein complexes and cellular compartments, thiol groups within a polar environment or surface-oriented domains are preferentially targeted. These include (i) enzymes associated with energy metabolism, (ii) components of the cytoskeleton, (iii) stress proteins and (iv) signaling proteins as recently defined by the redox proteomic profiling of Jurkat cells in response to treatment with H_2_O_2_ [21]. The cellular redox status has also been shown to play an important role in modulating effector functions of lymphocytes [7], [22] by altering cytokine production pattern and signal transduction pathways [7]. However, so far there is little known about general alterations within the protein expression profile of naïve and memory/effector T cells in response to treatment low doses of H_2_O_2_. Thus, the 2-DE-based proteomic profiling data presented in this study revealed that a significant number of the proteins that were previously defined as being sensitive to undergoing modifications in the presence of reactive oxygen species (ROS) can now also be classified as differentially expressed in response to exposure to 5 µM H_2_O_2_, at least in naïve and effector/memory CD45 T cells (Table 2, Table S1).

As representatively shown in Figure 2 and summarized in Table 1 the overall spot pattern defining the distinct T cell subsets plus minus treatment with low doses of H_2_O_2_ were quite consistent across the various labeling approaches. This also holds up for the overall regulation pattern in particular for the silver and the minimal DIGE labelings, both analyzed with the Proteomweaver software package, which strictly considers the regulation mode within the group of matched spots within the respective match set (Table 1, column 1 and 2). The differences documented in the regulation pattern defined with the Delta2D software package (Table 1, columm 3) are based on its different matching concept rather relying on a 100% spot matching algorithm. Thus, the total number of differentially expressed protein is significantly increased despite an overall largely consistent spot pattern.

A number of differentially expressed protein spots defined via the proteomic profiling of CD45RA^+^ vs. CD45RO^+^ T cells are linked to the energy metabolism and the maintenance of the redox capacity. Whereas increased expression levels of phosphoglycerate kinase 1 (PGK1), fructose-bisphosphate aldolase A (ALDO) and manganese superoxide dismutase (SODM) were detected in CD45RO^+^ T cells, CD45RA^+^ T cells displayed higher expression levels for the antioxidant thioredoxin (THIO), likely contributing to the previously defined superior ROS tolerance of this T cell subset. Moreover, the expression pattern for the latter targets is fully supported both by the qPCR (Figure 3A) and Western blot results (Figure 4). However, the main focus of this study was set on defining differences in the protein expression profile of naïve and effector/memory T cells in response to exposure to low doses of H_2_O_2_. Some of the oxidative stress regulated proteins in both cellular subsets, such as enzymes associated with the maintenance of the cellular redox status, with signaling pathways or with the shaping/reorganization of the cytoskeleton exert a high biologic variance as demonstrated by the 2-DE-based profiling results (Table 4), which was also confirmed at the transcriptional (Figure 3) and translational level (Table 5). The H_2_O_2_-mediated up-regulation of PRDX2 and GDIR1/GDIA1 in CD45RA^+^, but not in CD45RO^+^ T cells are consistent with the comparative transcriptomic expression profiling results (Table S1, Table 2 and Figure 4C), while the expression of SODM and GDIR2 appears to be rather regulated at the posttranscriptional and/or posttranslational level. This is further supported by stable transcript levels for SOD2 and GDIA2 and a heterogeneous regulation of the corresponding protein expression levels in CD45R0^+^ T cells (Table S1, Figure 3, Table 4). In addition, discrepancies exist in the regulation patterns of e.g. GSTO1, which is down-regulated at the transcriptional, but predominantly up-regulated at the protein expression level in CD45RA^+^ T cells in response to oxidative stress exist (Table S1, Figure 3, Table 4). Although the underlying molecular mechanisms of the observed counter-regulations in response to oxidative stress have still to be determined, the obtained data suggest a lack of hierarchy and no common regulation pattern in particular for targets displaying antioxidant activity. This leads to the assumption that T cells might rather randomly use their pools of antioxidants to counteract oxidative stress conditions.

At the molecular level oxidative stress mediated by e.g. ROS can either induce apoptotic or necrotic cell death or stimulate autophagy to counter ROS as a cellular survival mechanism [16], [23]. Oxidation of intracellular and/or extracellular thiol systems might cause a dysregulation of the Ca^2+^ homeostasis, an increased permeability of the mitochondrial membrane as well as the transition and activation of the apoptosis signal-regulating kinase 1 (ASK-1, MAP/ERK kinase kinase 5). H_2_O_2_ predominantly oxidizes thiol groups rather than leading to non-targeted crosslinking of cellular macromolecules. As a result of reversible redox reactions or by undergoing posttranslational modifications, such as glutathionylation, acetylation, nitrosylation, sulfhydration or metal binding, Cys residues are not only involved in controlling catalytic sites, but also function as sensors or modulators of signal transduction processes, in particular in response to oxidative stress, thereby modulation enzymes associated with energy metabolism, anti-oxidative stress and cellular redox control [16], [23]. Thus, some of the conserved Cys residues frequently found in cytoskeletal proteins might even serve as redox-sensors [24]. Under oxidative stress the cytoskeleton and in particular actin is prone to undergo massive oxidation and/or crosslinking with many proteins [25]. Hence, redox sensing via oxidation of Cys residues might be relevant not only for the coordination and organization of the protein machinery, but also serve to control changes regarding cell morphology by regulating the distribution and interaction of proteins attached to the cytoskeleton.

There is increasing evidence that in response to ROS treatment autophagy can lead to the induction of or protection from apoptosis in cells, which is likely dependent on the extent and duration of the exposure as well as on the surrounding cellular environment [26]. The proteomic profiling of Jurkat T-leukemia cells either left untreated or treated with the histone deacetylase inhibitor suberoylanilide hydroxamic acid (SAHA) known to induce ROS and subsequently autophagy demonstrated an altered expression pattern of enzymes involved in the energy metabolism, the cellular redox control and anti-oxidative proteins [16]. A similar response pattern was also found in hepatoma or lung carcinoma cells challenged with the carcinogen benzo(a)pyrene or chlorinated benzenes, respectively, leading to massive oxidative stress [27], [28]. This data suggest that massive oxidative stress might rather trigger common response pattern, almost independent from the initiating stimuli.

Since the H_2_O_2_ concentration as well as the exposure time used for the current proteomic profiling study were intended to minimize pro-apoptotic/autophagy effects only a very limited overlap of targets was observed when compared to that of the aforementioned profilings suggesting that exposure to low concentration of H_2_O_2_ rather triggers T cell anergy than lasting cell damage without critically disturbing, the intracellular redox balance.

The H_2_O_2_-mediated down-regulation of THIO/TRX1, a major intracellular anti-oxidative protein in CD45RO^+^ T cells in response to oxidative stress (Table S1, Figure 3C, Table 5) might explain to some extent their higher sensitivity to ROS when compared to CD45RA^+^ T cells [12] and to regulatory T cells (Tregs), which express and secrete higher levels of THIO [29]. However, the benefits of introducing transgenic TRX1 into this particular T cell subset remains to be evaluated in further studies. Overexpression of THIO is linked to cancer and angiogenesis as well as to T cell proliferation. In response to oxidative stress THIO can be either translocated into the nucleus [30] or secreted, thereby enhancing NF-kB transcriptional activities or counteracting extracellular stress via its scavenger function. Moreover, the oxidation of specific Cys residues proximal to the active site (Cys-32, Cys-35) within the THIO molecule is linked to the induction of apoptosis via mitogen-activated protein kinase (MAPK)/ERK kinase kinase 5 (ASK-1) [31], whereas the oxidation of another redox-sensing dithiol/disulfide motif (Cys-62, Cys-69) more distal to the active site appears to be rather involved in transient inhibition of THIO activity under oxidative stress conditions by controlling the reduction of the disulfide bridge between Cys-32 and Cys-35 thereby increasing the sensing and transmission of of oxidative signals [32]. Thus, the site of oxidation seems to be crucial for the cellular function of THIO.

GSTO1 catalyses the conjugation of GSH with other molecules and serves as an intermediate in detoxification processes. The relative intracellular redox potential is reflected by the ratio of reduced versus oxidized glutathione (GSH/GSSG), which has a strong impact on the proliferative capacity of challenged cells. The proliferation rate of mitogen-treated T cells is not only correlated with the intracellular redox status, but also depends on adequate glutamine supply [33]. The intracellular GSH level is also strongly influenced by the relative glutamic acid supply, another constituent amino acid of GSH [34] or the availability of extracellular GSH as shown for various epithelial tumor cells [35]. Moreover, the expression level of THIO based on its catalytic activity of reducing oxidized cysteine groups has a profound impact on the cellular redox status by balancing the GSH/GSSG ratio thereby supporting cell proliferation and inhibition of apoptosis [36]. Also, the predominantly observed up-regulation of SODM in CD45RA^+^ T cells in response to low doses of H_2_O_2_ (Figure 3C, Table 5) might be involved in counteracting mild oxidative stress, in line with its ROS and hypoxia-mediated induction [20]. Likewise, the transduction of T cells with CAT protects against oxidative stress [30]. In addition to SODM, GSTO1, PRDX2 and THIO also SH3 domain-binding glutamic acid-rich-like protein 3 (SH3L3) and the main producer of NADPH glucose-6-phosphate 1-dehydrogenase (G6PD) involved in maintaining the cellular redox status were also found to be differentially expressed in the distinct T cell subsets (Table S1).

Redox proteome-based profiling strategies such as redox Western blotting or two-dimensional liquid chromatography separation in combination with tandem mass spectrometry (2D-LC-MSMS) demonstrated that many of the proteins defined as differentially expressed via the 2-DE-based expression profiling of distinct T cell subsets in the presence and absence of 5 mM H_2_O_2_ can also undergo redox changes under distinct biological conditions depending on the redox status within a given cellular compartment [37], [38] as well as within the extracellular space thereby modulating distinct cellular and biologic processes [39], [40], [41]. The data presented in this report extend the knowledge by demonstrating that many of the targets that are sensitive to undergo modifications under oxidative stress were also differentially expressed (Table S1, Table 2). In regard to signal transduction proteins mainly components linked to the modulation of apoptosis and focal adhesion were altered. Whereas annexin A5 (ANXA5) and GDIR2 are thought to counteract the induction of apoptosis supporting the finding that exposure to low doses of H_2_O_2_ hardly triggers apoptotic cell death in both T cell subsets, thrombospondin 1 (TBSP1), integrin alpha-IIb (ITA2B), LIMS1 and the growth factor-related protein 2 (GRB2) can be associated with the formation of focal adhesion points. In line with the differential expression of signaling molecules associated with focal adhesion the adapter proteins alpha-actinin-1 (ACTN1), filamin A (FLNA), talin (TLN1) and vinculin (VINC), serving as cytoskeletal anchors, also showed altered expression pattern in response to exposure to low doses of H_2_O_2_, indicating that oxidative stress has an impact on cell morphology. Further evidence is provided by the finding that PROF1, cofilin-1 (COF1), Rho-GTPase-binding and Rho protein signal transduction proteins as well as plastin-2 (PSPL), which can be also linked to T cell activation and thus morphologic changes were also differentially expressed in response to mild oxidative stress (Table S1). This is further supported by the observed differential expression pattern of cytoskeletal proteins, like various tubulins (tubulin beta chain (TBB5), tubulin alpha-1A chain (TBA1A), tubulin alpha-4A chain (TBA4A), tubulin alpha-8A chain TBA8), tropomyosins (tropomyosin alpha-1 chain (TPM1), tropomyosin beta chain (TPM2), tropomyosin alpha-3 chain (TPM3), tropomyosin alpha-4 chain (TPM4) and actin-related proteins (actin-related protein 2/3 complex subunit 2 (ARPC2), actin-related protein 3 (ARP3) are modulated in response to H_2_O_2_ treatment or between the distinct T cell subsets. In addition, a number of cellular stress proteins and chaperones, like the heat shock proteins (HSP) heat shock protein HSP 90-alpha (HS90A), GRP75, GRP78, HSP7C and protein disulfide isomerase A3 (PDIA3) showed an altered expression pattern indicating that the cells encountered mild cellular/oxidative stress (Table S1).

Taken together, the differentially expressed proteins defined by exposing naïve and effector/memory T cells to low doses of H_2_O_2_ can in part be directed linked to an antioxidant-mediated response, yet the observed variances argue against a strict hierarchical order in the usage of cellular antioxidants. In addition, the presented profiling data suggest that mild oxidative stress might affect the modulation/reshaping of the cytoskeleton. The mechanisms underlying the observed differences between the mRNA and protein levels of some of the verified targets might be related to the relatively short exposure time to H_2_O_2_ of 3 hours. However, despite this limitation the panel of differentially expressed proteins defined by applying the various staining/labeling strategies provides novel insights into the cellular response of T cell subsets to oxidative stress.

## Materials and Methods

### Isolation of immune cells

The experiments in this manuscript were performed with lymphocytes isolated from peripheral blood mononuclear cells (PBMC), either obtained from the Karolinska Hospital Blood Central or from the Department of Transfusion Medicine of the University Hospital of the Martin Luther University Halle-Wittenberg. Since all of the blood samples were obtained from anonymous healthy donors no informed consent is needed. Following density gradient-based Ficoll-Paque separation (Amersham Biosciences, Sunnyvale, CA) CD3^+^ T cells were purified using a Pan T cell isolation kit. Short-term cultures produced by the authors from such preparations as part of the present study served as a resource for the specific isolation of untouched naïve CD45RA^+^ and memory CD45RA^−^ CD3^+^ T cell subpopulations (purity >95%) by depletion-steps using anti-CD45RA/RO-specific microbeads and a negative selection strategy, respectively. All antibody-conjugated microbeads and separation columns for the cell isolations were purchased from Miltenyi Biotec (Auburn, CA). For according purity controls, cells were stained with mouse monoclonal antibodies (mAb) directed against human CD3-FITC (UCHT1), CD45RA-APC (HI100) and CD45RO-APC (UCHL1) (BD Biosciences, San Jose, CA) and analyzed on a FACS Calibur flow cytometer (BD Biosciences).

### Cell culture and induction of oxidative stress

Cells at a density of 1×10^6^ cells/mL were cultured in serum-free AIM-V medium (Invitrogen, Carlsbad, CA) for 3 hours in the presence or absence of 5 µM H_2_O_2_.,harvested and washed in phosphate buffered saline (PBS) before cell pellets were stored at −80°C until further use. As a control of H_2_O_2_ bioactivity, cells were cultured for 24 hours in the presence of 5 µM H_2_O_2_. The significant cell death was assessed and confirmed by 7-amino-actinomycin D (7-AAD) staining and forward-to-side-scatter profile..

### Two-dimensional gel electrophoresis (2-DE) and difference gel electrophoresis (DIGE)

Naïve (CD45RA^+^) and memory T cells (CD45RO^+^) either left untreated or treated with 5 µM H_2_O_2_ were initially subjected to 2-DE-based protein expression profilings relying on sensitive silver stainings as previously described [42]. In addition, the biological variances within the respective expression profiles were assessed by performing further comparative expression profilings applying either minimal or saturating labeling difference gel electrophoresis (DIGE) strategies according to the manufacturer's instructions (Amersham Biosciences, Freiburg, Germany) as recently described [43], [44].

### Image analysis

Image analysis was carried out either with the Proteomweaver (Version4.0, Bio-Rad GmbH, Munich, Germany, silver staining, minimal DIGE labeling) or the Delta2D (Version 4.0, Decodon GmbH, Greifswald, Germany; saturating DIGE labeling) software packages according to the manufacturer's guidelines. Proteins displaying a regulation factor ≥2.0 (silver staining), ≥1.5 (DIGE labeling) or exhibiting a restricted expression pattern in one of the given conditions were classified as differentially expressed and were selected for subsequent microanalytical characterization.

### Mass spectrometry and data mining

Due to the very limited sample material available, preparative gels were run with samples representing unfractionated T cells isolated via Ficoll gradients from peripheral blood mononuclear cells (PBMC) obtained from healthy donors. Therefore, the analysis was only restricted to those spots, which could be matched between the profiles of the crude and fractionated T cell subsets. The preparative gels were loaded with 750 µg total lysate, the spots of interest were subsequently in gel digested and subjected to mass spectrometry, using the matrix-assisted laser-desorption/ionization time of flight (MALDI-TOF) instrument Voyager DE™ PRO (Applied Biosystems, Forster City, CA, USA) as previously described [43]. Database searches were performed employing the MASCOT software package (Matrix Science, London, UK).

The data sets of the proteins identified were linked to the UniProtKB/Swiss-Prot identities (UniProt Knowledgebase Release 2011_12, Dec 14, 2011) and gene ontology (geneontology.org). Based on this information the protein families, chromosomal and cellular localizations of differentially expressed proteins as well as their function were assigned to each protein.

### qRT-PCR analysis

Total cellular RNA was extracted using the RNeasy mini kit (QIAGEN, Valencia, CA) according to the manufacturer's instructions. Subsequently cDNA was prepared with the iScript cDNA synthesis kit (Bio-Rad, Hercules, CA) following the manufacturer's instructions before the transcript levels were quantified by qRT-PCR (SYBR Green Supermix; ABI PRISM 7500, Applied Biosystems). Relative gene expression was determined by normalizing the expression level for each target gene to the expression level of beta-actin. Primer sequences used for amplifications are summarized in Table S2.

### Western blot analysis

Total protein lysates generated from either untreated or H_2_O_2_-treated (5 µM, exposed for 3 hours) T cells (CD45RA^+^, CD45RO^+^) were separated by Tris-Tricine gels (16%T/2.5%C), transferred to PVDF membrane (Roche Diagnostics, Mannheim, Germany) and the membranes thereafter blocked as previously described [42], [45]. The membranes were subsequently serially incubated with target-specific antibodies directed against thioredoxin (THIO) (ab16835, Abcam plc, Cambridge, UK; dilution 1∶2000), glutathione S-transferase O1 (GSTO1) (LS-C15465, Life Span Biosciences Inc, Seattle, WA, USA; dilution 1∶2000), peroxiredoxin 2 (PRDX2) (ab15572, Abcam; dilution 1∶10000), profilin (PROF1) (sc-30521, Santa Cruz Biotechnology Inc, Heidelberg, Germany; dilution 1∶500), LIMS1 (Pinch 1) (sc-47912, Santa Cruz), Rho GDP-dissociation inhibitor 1 (GDIR1) (10509-1-Ig, Proteintech Europe Ltd, Manchester, UK; dilution 1∶500), Rho GDP-dissociation inhibitor 2 (GDIR2) (ab15198, Abcam; dilution 1∶200) or superoxide dismutase 2 (SODM) (LF-MA0030, Lab Frontier Life Science, Seoul, Korea; dilution 1∶2000). Staining of the membranes with the beta-actin-specific mAb AC15 (ab6276; Abcam) served as a loading control. Following at least 3 washing steps in TBST the membranes were incubated with suited horseradish peroxidase (HRP)-conjugated secondary antibodies (P0217, P0260, P0449, DAKO, Hamburg, Germany; ab6877, Abcam), washed again at least 3 times in TBST prior to the visualization of the respective immunostainings using a chemiluminescent detection kit (LumiLight Western blot substrate, Roche, Germany) according to the manufacturer's recommendations. The quantification of the respective protein expression levels was determined by applying the advanced image data analyzer (AIDA) software package (Image analyser V. 4.11, raytest Isotopenmessgeraete GmbH, Berlin, Germany). Relative protein expression levels are given as arbitrary units by setting the peak values of corresponding beta-actin signals to 1.

## Supporting Information

Figure S1 **Purity of naïve and memory/effector T cell subpopulations.** Representative dot-plots of the isolated naïve CD3^+^CD45RA^+^ (left panel) and memory/effector CD3^+^CD45RO^+^ (right panel) T cell subsets are shown as analyzed by flow cytometry.
(PDF)

Figure S2 **Gene ontology (GO)-based classification for the panel of differentially expressed proteins in regard to their cellular function und localization proteins.** The pie charts display the classification of the subset of differentially expressed proteins into 10 functional categories (A) and into 9 distinct cellular compartments (B). The pool of proteins is comprised of 56 distinct protein identities. The distribution frequencies in regard to the specified categories within the given chart pie are indicated in percentage of the total number of entries. For each pie chart a cut-off value of 5% was set. Therefore the functions, e.g. immune response (4%), adhesion, expression control and proliferation (2% each) (Figure A) and the compartments, e.g. membrane, Golgi apparatus (2% each), nucleus and ER (4% each) (Figure B) were collected in the category “other”, respectively.
(PDF)

Table S1 **List of identified proteins as defined in the various match sets.** Table S1 provides a brief summary of the mass spectrometry results along with the full list proteins defined as differentially expressed across the various match sets. Whereas the protein and entry names of up-regulated targets are listed in Table S1 A the corresponding data for down-regulated targets are listed in Table S1 B.
(DOC)

Table S2 **Primers used for RT-PCR analysis.** Table S2 provides the full list of primers applied in the verification experiments. The panel of verified target genes is specified by the respective entry name, NCBI reference sequence code along with the full information on the used forward and reverse primer sequences.
(DOC)
