# Supplementary figures and images for: Comparative Expression Profiling of Distinct T Cell Subsets Undergoing Oxidative Stress
Source: PLoS One. 2012 Jul 20;7(7):e41345. doi: 10.1371/journal.pone.0041345 (PMC3401147; doi:10.1371/journal.pone.0041345)

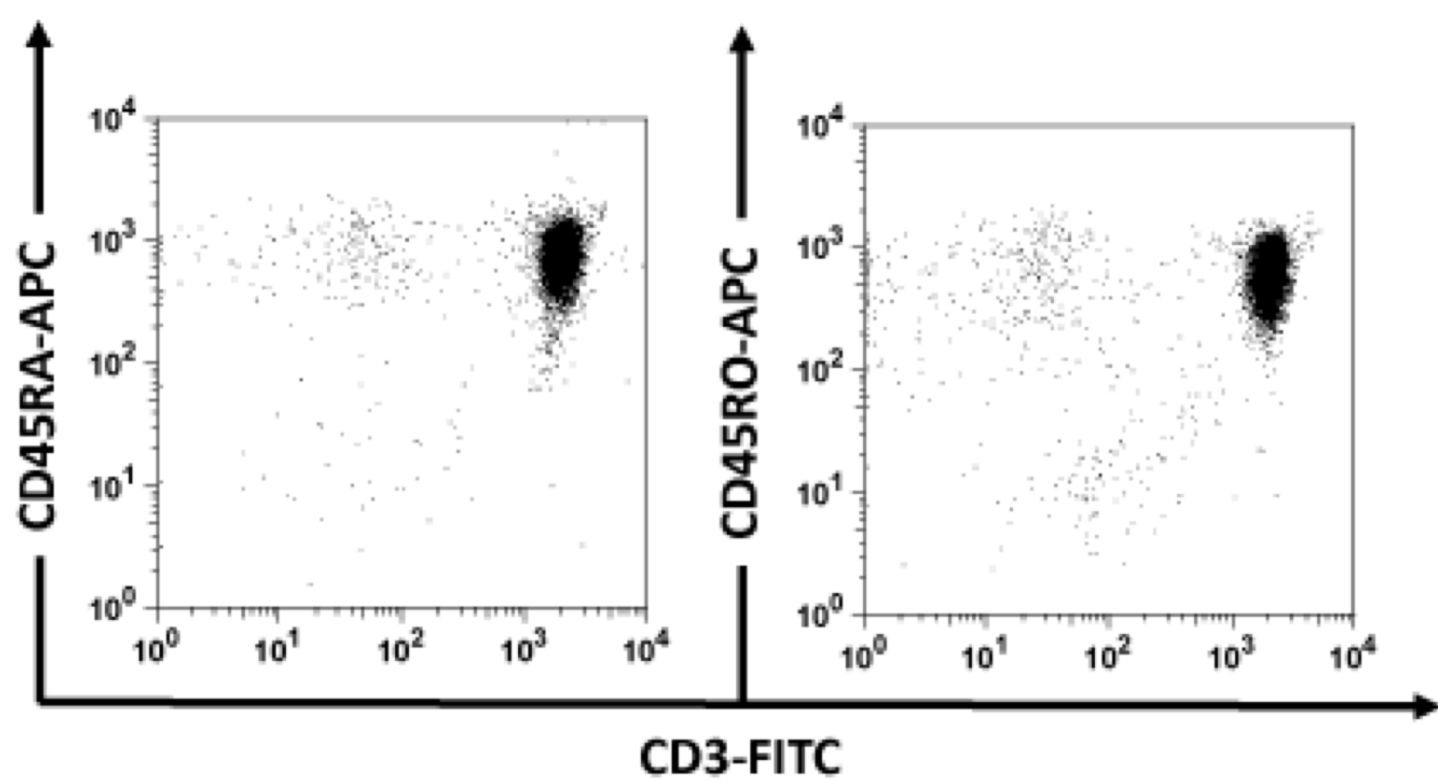

Supplement: Figure S1 — Purity of naïve and memory/effector T cell subpopulations. Representative dot-plots of the isolated naïve CD3+CD45RA+ (left panel) and memory/effector CD3+CD45RO+ (right panel) T cell subsets are shown as analyzed by flow cytometry. (PDF) [file pone.0041345.s001.pdf]

A

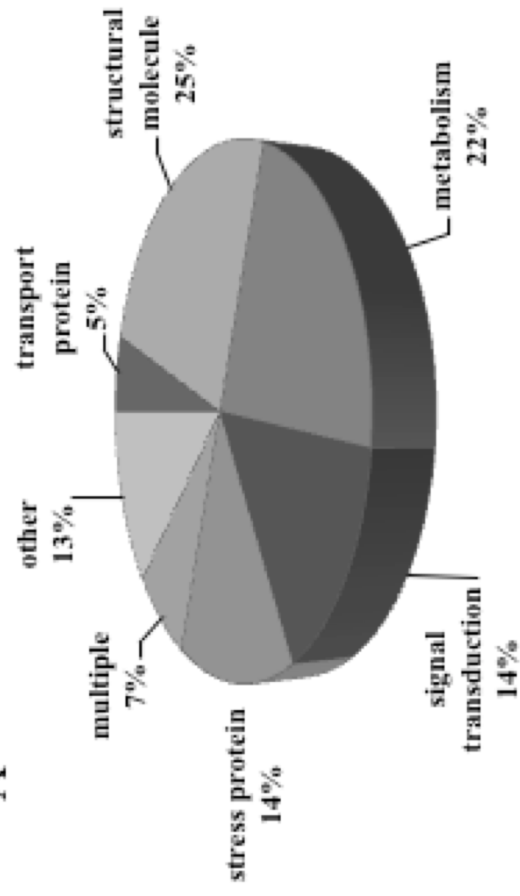

B

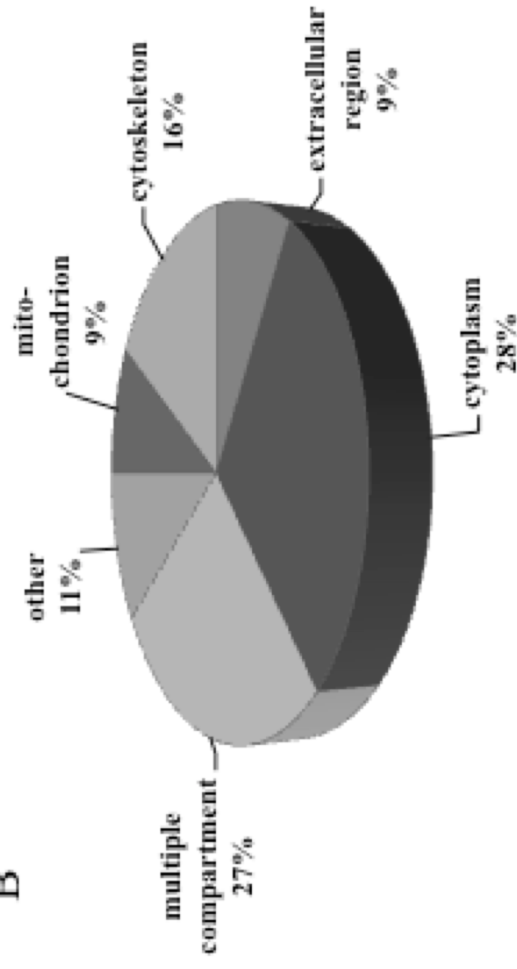

Supplement: Figure S2 — Gene ontology (GO)-based classification for the panel of differentially expressed proteins in regard to their cellular function und localization proteins. The pie charts display the classification of the subset of differentially expressed proteins into 10 functional categories (A) and into 9 distinct cellular compartments (B). The pool of proteins is comprised of 56 distinct protein identities. The distribution frequencies in regard to the specified categories within the given chart pie are indicated in percentage of the total number of entries. For each pie chart a cut-off value of 5% was set. Therefore the functions, e.g. immune response (4%), adhesion, expression control and proliferation (2% each) (Figure A) and the compartments, e.g. membrane, Golgi apparatus (2% each), nucleus and ER (4% each) (Figure B) were collected in the category “other”, respectively. (PDF) [file pone.0041345.s002.pdf]
