# Supplementary material for: Comparative Expression Profiling of Distinct T Cell Subsets Undergoing Oxidative Stress
Source: PLoS One. 2012 Jul 20;7(7):e41345. doi: 10.1371/journal.pone.0041345 (PMC3401147; doi:10.1371/journal.pone.0041345)
Supplement: Table S1 — List of identified proteins as defined in the various match sets. Table S1 provides a brief summary of the mass spectrometry results along with the full list proteins defined as differentially expressed across the various match sets. Whereas the protein and entry names of up-regulated targets are listed in Table S1 A the corresponding data for down-regulated targets are listed in Table S1 B. (DOC) [file pone.0041345.s003.doc]

**Table S1: List of identified proteins as defined in the various match sets**

| **A** |  | **CD45RA^+^  treated/untreated up-regulated** | | | **CD45RO^+^ treated/untreated up-regulated** | | | **CD45RO^+^ vs. CD45RA^+^ up-regulated** | | | **CD45RO^+^ H_2_O_2_^-^treated vs. CD45RA^+^ H_2_O_2_^-^treated up-regulated** | | |
| --- | --- | --- | --- | --- | --- | --- | --- | --- | --- | --- | --- | --- | --- |
|  |  | spots analysed | spots identified | single targets | spots analysed | spots identified | single targets | spots analysed | spots identified | single targets | spots analysed | spots identified | single targets |
|  |  | 34 | 30 | 23 | 24 | 20 | 17 | 33 | 30 | 20 | 7 | 7 | 5 |
| protein |  | **14-3-3 zeta/delta (1433Z) 60 kDa heat shock protein, mitochondrial (CH60)** Actin beta (ACTB)/gamma (ACTG) **Actin-related protein 2/3 complex subunit 2 (ARPC2) Alpha-actinin-1 (ACTN1)** Alpha-actinin-1 (ACTN1) **Alpha-enolase (ENOA) Alpha-enolase (ENOA) Coagulation factor XIII A chain (F13A) Coagulation factor XIII A chain (F13A)** Coronin-1A (COR1A) Cytokeratin 10 (K1C10) Fibrinogen beta chain (FIBB) Filamin-A (FLNA) Gelsolin (GELS) **Glutathione S-transferase omega-1 (GSTO1) Heat shock cognate 71 kDa protein (HSP7C)** Heat shock protein HSP 90-alpha (HS90A) **Integrin alpha-IIb (ITA2B) Peroxiredoxin-2 (PRDX2) Plastin-2 (PLSL) Rho GDP-dissociation inhibitor 1 (GDIR1) Stress-70 protein, mitochondrial (GRP75) Talin-1 (TLN1) Thrombospondin-1 (TSP1) Transaldolase (TALDO1) Tropomyosin alpha-1 chain (TPM1) Tropomyosin alpha-3 chain (TPM3) Tropomyosin alpha-4 chain (TPM4)** Tubulin alpha-4A chain (TBA4A) Vimentin (VIME) **Vinculin (VINC)** | | | Actin beta (ACTB)/gamma (ACTG) **Actin-related protein 3 (ARP3)** Annexin A6 (ANXA6) **ATP synthase subunit beta, mitochondrial (ATPB) ATP synthase subunit d, mitochondrial (ATP5H) Cofilin-1 (COF1) Fibrinogen beta chain (FIBB) Fibrinogen gamma chain (FIBG) Fibrinogen gamma chain (FIBG)** Filamin-A (FLNA) **Heat shock cognate 71 kDa protein (HSP7C) L-lactate dehydrogenase B chain (LDHB) Protein disulfide-isomerase A3 (PDIA3) Rho GDP-dissociation inhibitor 2 (GDIR2) Serum albumin (ALBU)** Stress-70 protein, mitochondrial (GRP75**) Tubulin alpha-4A** **chain (TBA4A)** **Tubulin beta chain (TBB5) UDP-glucose-1 phosphate uridyltransferase (UGPA) Vinculin (VINC)** Vinculin (VINC) | | | **78 kDa glucose-regulated protein (GRP78)** Actin beta (ACTB)/gamma (ACTG) Alpha-actinin-1 (ACTN1) **Alpha-enolase (ENOA) Annexin A5 (ANXA5) ATP synthase subunit beta, mitochondrial (ATPB)** Calnexin (CALX) Colorectal mutant cancer protein (CRCM) Cytokeratin 1 (K2C1) Cytokeratin 2e (K22E) Cytokeratin 9 (K1C9) Cytokeratin 10 (K1C10) **Fibrinogen beta chain (FIBB) Fibrinogen beta chain (FIBB) Fructose-bisphosphate aldolase A (ALDOA) Fructose-bisphosphate aldolase A (ALDOA** Glucose-6-phosphate 1-dehydrogenase (G6PD) **Heat shock cognate 71 kDa protein (HSP7C) Heat shock cognate 71 kDa protein (HSP7C) LIM and senescent cell antigen-like-containing domain protein 1 (LIMS1, PINCH1) Phosphoglycerate kinase 1 (PGK1) SH3 domain-binding glutamic acid-rich -ike protein 3 (SH3L3) Stress-70 protein, mitochondrial (GRP75) Superoxide dismutase (Mn), mitochondrial (SODM) Talin-1 (TLN1)** T-complex protein 1 subunit delta (TCPD) **Tubulin alpha 8 (TBA8)** Tubulin alpha-4A chain (TBA4A) Tubulin alpha-1A chain (TBA1A) **Vinculin (VINC)** | | | Actin beta (ACTB)/gamma (ACTG) **ATP synthase subunit d, mitochondrial (ATP5H) Glutamate dehydrogenase 1, mitochondrial (DHE3) Protein disulfide-isomerase A3 (PDIA3) Transketolase (TKT) Triosephosphate isomerase (TPIS)** | | |
|  |  |  | | |  | | |  | | |  | | |

| **B** | | **CD45RA^+^ treated/untreated downregulated** | | | | | | | | | | **CD45RO^+^ treated/untreated downregulated** | | | | | | | **CD45RO^+^ vs. CD45RA^+^ downregulated ^+^** | | | | | | | | | **CD45RO^+^ H_2_O_2_^-^treated vs. CD45RA^+^ H_2_O_2_^-^treated downregulated** | | | | | | | | | | |  | | |
| --- | --- | --- | --- | --- | --- | --- | --- | --- | --- | --- | --- | --- | --- | --- | --- | --- | --- | --- | --- | --- | --- | --- | --- | --- | --- | --- | --- | --- | --- | --- | --- | --- | --- | --- | --- | --- | --- | --- | --- | --- | --- |
|  |  | | | spots analysed | | spots identified | | single targets | | | spots analysed | | | spots identified | | | single targets | | spots analysed | | | spots identified | | single targets | | | spots analysed | | | | | | spots identified | | single targets | | | | | |  |
|  | | | 9 | | | | 8 | | | 7 | | | 17 | | | 12 | | 110 | | | 5 | | 3 | | 3 | | | 3 | | | | 3 | | | | 1 | |  | | | |
| protein | | Alpha-actinin-1 (ACTN1) **Calreticulin (CALR) Coactosin-like protein (COTL1) Fibrinogen beta chain (FIBB) LIM and senescent cell antigen-like-containing domain protein 1 (LIMS1, PINCH1) Pyruvate kinase isoenzymes M1/M2 (KPYM) Tropomyosin alpha-1 chain (TPM1) Tropomyosin beta chain (TPM2)** Vimentin (VIME) | | | | | | | | | | Actin beta (ACTB)/gamma (ACTG) **Actin-related protein 2/3 complex subunit 2 (ARPC2) ATP synthase subunit d, mitochondrial (ATP5H) Filamin-A (FLNA**) **Fructose-bisphosphate aldolase A (ALDOA) Growth factor receptor-bound protein 2 (GRB2) Heat shock protein HSP 90-alpha (HS90A)** Phosphoglycerate kinase 1 (PGK1) **SH3 domain-binding glutamic acid-rich -ike protein 3 (SH3L3) Superoxide dismutase (Mn), mitochondrial (SODM) Transaldolase (TALDO) Tubulin beta chain (TBB5)** | | | | | | | | | **Profilin 1 (PROF1) Thioredoxin (THIO) Tropomyosin alpha-4 chain (TPM4)** | | | | | | | | Cytokeratin 10 (K1C10) Ribonuclease inhibitor (RNH1) **Tubulin beta chain (TBB5)** | | | | | | | |  | | | | |
|  | |  | | | | | | | | | |  | | | | | | | |  | | | | | | | | | |  | | | | | | | | | | | |
|  | |  | | |  | | | |  | | |  | | |  | | |  | |  | | |  | | |  | | | | |  | | |  | | | |  | |  | |

bold lettering indicates single target spots
