# Supplementary material for: Comparative Expression Profiling of Distinct T Cell Subsets Undergoing Oxidative Stress
Source: PLoS One. 2012 Jul 20;7(7):e41345. doi: 10.1371/journal.pone.0041345 (PMC3401147; doi:10.1371/journal.pone.0041345)
Supplement: Table S2 — Primers used for RT-PCR analysis. Table S2 provides the full list of primers applied in the verification experiments. The panel of verified target genes is specified by the respective entry name, NCBI reference sequence code along with the full information on the used forward and reverse primer sequences. (DOC) [file pone.0041345.s004.doc]

**Table S2: Primers used for RT-PCR analysis**

| **target gene** | **NCBI reference sequence** | **primer sequence** |
| --- | --- | --- |
|  |  |  |
| ACTB | NM_001101.3 | forward 5’-AGGCACCAGGGCGTGAT-3’ reverse 5’-TCGTCCCAGTTGGTGACGAT-3’ |
| GSTO1 | NM_004832.1 | forward 5’-GGAAAACAGTCAGGGTCAGC-3’ reverse 5’-CAGCTTCTTCCCTGGGTATG-3’ |
| LIMS1 | NM_004987.3 | forward 5’-TGGACATCGCCATTATGAGA-3’ reverse 5’-GCAGGTAAGCGTGCTCCC-3’ |
| SOD2 | NM_001024465.1 | forward 5’-CTTCAGCCTGCACTGAAGTTCAAT-3’ reverse 5’-CTGAAGGTAAGCGTGCTCCC-3’ |
| PFN1 | NM_005022.2 | forward 5’-CGAGAGCAGCCCAGTAGCAGC-3’ reverse 5’-ACCAGGACACCCACCTCAGCTG-3’ |
| PRDX2 | NM_005809.4 | forward 5’-TGACACGATTAAGCCCAACGT-3’ reverse 5’-GCACAAGCTCACTATCCGTTAGC-3’ |
| GDIA1 | NM_004309.3 | forward 5’-AACCGAGAGATAGTGTCCGGC-3’ reverse 5’-TCTTGACGCCTTTCCTGTACG-3’ |
| GDIA2 | NM_001175.4 | forward 5’-AGCAACTAGAGGCCAGAAAATGG-3’ reverse 5’-CTGGAACCTGAGTCAAAGACCTG-3’ |
| TRX1 | NM_003329.2 | forward 5’-ACGCTGCAGGTGATAAAC-3’ reverse 5’-CTGACAGTCATCCACATCTAC-3’ |
